# Changes in socioeconomic differentials in old age life expectancy in four Nordic countries: the impact of educational expansion and education-specific mortality

**DOI:** 10.1007/s10433-022-00698-y

**Published:** 2022-04-15

**Authors:** Linda Enroth, Domantas Jasilionis, Laszlo Németh, Bjørn Heine Strand, Insani Tanjung, Louise Sundberg, Stefan Fors, Marja Jylhä, Henrik Brønnum-Hansen

**Affiliations:** 1 Faculty of Social Sciences (Health Sciences) and Gerontology Research Center, Tampere University, Tampere, Finland; 2 Max Planck Institute for Demographic Research, Rostock, Germany; 3 Department of Physical Health and Ageing, Norwegian Institute of Public Health, Oslo, Norway; 4 The Norwegian National Centre for Ageing and Health, Vestfold Hospital Trust, Tønsberg, Norway; 5 Department of Geriatric Medicine, Oslo University Hospital, Oslo, Norway; 6 Aging Research Center, Karolinska Institutet, Stockholm, Sweden; 7 Stockholm University, Stockholm, Sweden; 8 Department of Public Health, University of Copenhagen, Copenhagen, Denmark

**Keywords:** Longevity, Socioeconomic status, Trend, Decomposition

## Abstract

Overall progress in life expectancy (LE) depends increasingly on survival in older ages. The birth cohorts now reaching old age have experienced considerable educational expansion, which is a driving force for the social change and social inequality. Thus, this study examines changes in old age LE by educational attainment in the Nordic countries and aims to find out to what extent the change in national LEs is attributable to education-specific mortality and the shifting educational composition. We used national register data comprising total 65 + populations in Denmark, Finland, Norway and Sweden to create period life tables stratified by five-year age groups (65–90 +), sex and educational attainment. Difference in LE between 2001 and 2015 was decomposed into the contributions of mortality changes within each educational group and changes in educational composition. Increasing LE at all ages and in all educational groups coincided with persistent and growing educational inequalities in all countries. Most of the gains in LE at age 65 could be attributed to decreased mortality (63–90%), especially among those with low education, the largest educational group in most countries. The proportion of the increase in LE attributable to improved education was 10–37%, with the highest contributions recorded for women in Norway and Sweden. The rising educational levels in the Nordic countries still carry potential for further gains in national LEs. However, the educational expansion has contributed to uneven gains in LE between education groups, which poses a risk for the future increase of inequalities in LE.

**Supplementary Information:**

The online version contains supplementary material available at 10.1007/s10433-022-00698-y.

## Introduction

Socioeconomic inequalities in health and mortality are well established and have received extensive research attention in Western countries. Those with higher education, non-manual occupations, or high assets live longer and healthier lives than those with lower social and economic assets (WHO 2010). As life expectancy (LE) is one of the key indicators of population health, the recent period (2014–2015) of stalling or decreasing LE across several high-income countries (Ho and Hendi 2018) has prompted increasing interest in the mechanisms affecting LEs. At the current stage of the epidemiological transition where most deaths occur in old age, changes in LE are increasingly attributable to older age groups (Martikainen et al. 2001; Deboosere et al. 2009).

In several countries, the birth cohorts that experienced educational expansion are now reaching older ages. While most studies on socioeconomic inequalities in LE report gains over time in all socioeconomic groups, recent research suggests declines in older age LE among people with less than high school education and among those without any formal education in the US and Belgium (Deboosere et al. 2009; Hendi 2015, 2017). The changing educational structure contributes to the overall changes in LE and may lead to changes in the magnitude of socioeconomic inequalities. This study examines changes in old age LE by educational attainment and aims to find out to what extent changes in national LEs in the Nordic countries of Denmark, Finland, Norway, and Sweden can be attributed to education-specific mortality and to changes in educational composition.

Of the Nordic countries, the legislation for compulsory education was first established in Denmark (1814) and last in Finland (1921). Mass participation in primary and lower secondary education (ISCED 1–2) started earlier in Denmark and Norway compared with Finland and Sweden. However, despite the different starting points, all the Nordic countries experienced considerable educational expansion from the early norm of six to seven years compulsory education in the cohorts born in 1920–1960. For instance, the share of those with at least upper secondary education (ISCED 3) increased from less than 40% to more than 80% in these cohorts (Ballarino et al. 2013). In the 1960s and 1970s, the Nordic countries introduced universal compulsory education of nine years. The development of universal education was driven by ambitions to improve the general welfare of the population, increase economic efficiency, reduce educational inequality and promote and advance social mobility. In this sense educational expansion can be seen as part of a wider social policy drive to improve social welfare. Education not only increases knowledge and shapes behaviours, but it also provides formal qualifications, which influence future opportunities for employment and earnings (Lahelma et al. 2004; Montez et al. 2019). Therefore, changes in the educational structure have far-reaching implications for social inequality.

The proposed mechanisms through which educational attainment is associated with health and mortality are multifaceted and arise from unevenly distributed resources. The knowledge, norms and shared values adopted in higher education are likely conducive to healthier lifestyles such as physical activity, a healthy diet and smoking cessation (Laaksonen et al. 2008; Pampel et al. 2010). Furthermore, it has been shown that health literacy in terms of knowledge, motivation and competences to access, understand, appraise, and apply health information is weaker among those with low education, low social status and financial deprivation (Sørensen et al. 2015). Timely access to health care, which is critical for early diagnosis and a better prognosis, is associated with care-seeking behaviour and communications skills, but also depends on the availability and costs of care, which implies that several socioeconomic health-related factors in combination with the welfare system characteristics are likely to generate and maintain health inequalities. In addition to causal explanations, the associations between education and health have been explained by reference to health selection and indirect selection. The former suggests that people with poor health are less likely to achieve higher education, and the latter that the association may be confounded by individual traits such as IQ and personality, which are conducive to both education and health (Montez et al. 2019).

Research has consistently shown that people with higher education have longer LE than people with lower education (Valkonen and Martikainen 2006; Majer et al. 2011). However, there is a paucity of research into inequalities in LE in older populations. This is probably because life tables stratified by education are not routinely available and in most countries, information on the education is missing for the oldest age groups. The few existing register-based studies that cover total populations suggest that the educational gap in LE persist in older ages and that the gap between educational groups increased at age 85 in Norway between 1960 and 2009 (Kinge et al. 2015) and in Belgium between 1991 and 2004 (Deboosere et al. 2009). To our knowledge change in old age LE has not yet been decomposed into compositional changes in educational structure and education-specific mortality. However, we know from prior research that educational expansion has contributed to developments in LE and inequalities in LE at younger ages. Luy et al. (2019) showed that educational expansion contributed to a one-year increase in LE at age 30 in Denmark and Italy and to a half a year increase in the US between 1990 and 2010, with the relative contributions reaching around 20%. Also, Shkolnikov et al. (2006) reported that educational expansion had positive effect on LE at age 30 in four European countries from the mid-1980s to the late 1990s. Furthermore, it has been shown that in Finland, Sweden and Norway, educational expansion contributed substantially to increasing inequality in LE between 1971 and 2000 (Shkolnikov et al. 2012). Östergren et al. (2017) reported larger educational mortality inequalities in 18 European countries as a result of decreasing proportions of people with low education and increasing proportions of people with high education. Hence, educational expansion has contributed positively to LE, and at the same time may have increased inequalities in mortality between educational groups.

Health and social policies in the Nordic countries are aimed to supporting longevity and reducing social and economic inequalities (Lundberg et al. 2008). The Nordic countries share largely similar traditions and norms and are characterized as welfare states with universal health and social care systems. The ideal of the Nordic education model relies on compulsory basic education and equal opportunities for higher education, even though ways of organising universal education vary. However, despite the strong social welfare orientation, numerous studies have confirmed that socioeconomic status is a major determinant of health and mortality even in the Nordic countries (Enroth et al. 2019). Furthermore, in a European comparison, the Nordic countries show relatively large socioeconomic inequalities in mortality, sometimes referred to as the Nordic paradox (Mackenbach 2017).

In recent decades, old age LE has been on the rise in the Nordic countries, even though the pace of change has been uneven (Jørgensen et al. 2018) and international comparisons suggest increasing disadvantage in LE against the best-performing high-income countries because of the slower rate of improvement in old age mortality (Drefahl et al. 2014). In addition to slower LE development, the Nordic countries show less favourable trends in mortality inequalities compared with other parts of the Europe (Mackenbach et al. 2019). In sum, the Nordic countries have largely similar welfare models and education systems yet they have exhibited uneven trends in population ageing and are known for their relatively large socioeconomic inequalities in mortality. They therefore provide an interesting setting for studying the consequences of educational expansion for the LE development and inequality in LE.

Overall progress in national LE depends increasingly on survival in older ages. The birth cohorts now reaching old age have experienced considerable educational expansion, which has changed the sizes and probably the composition of educational groups in terms of social background and personal characteristics. The dynamics of the change in educational structure could be manifested in LE as follows: first, if health, cognitive ability and living conditions (e.g. wealth, social support) improved and enabled a larger part of the population to achieve higher education, the LE in the group with higher education would substantially increase. Second, if only access to higher education improved but those making the rise in educational level had less favourable living conditions, the social composition of those with higher education would change and could, at least temporarily, slowdown LE development in the larger but more heterogeneous group of people with higher education. Third, because of the increasing mobility into higher education, the group with only basic education would be reduced. As a consequence, those remaining in the group with basic education have likely become more negatively selected over time in terms of health and personal characteristics. In addition, their competitive position in the labour market has weakened over time, as the average educational level has increased in the population. Thus, LE development in this group would be slower or even stalling and would increasingly rely on the general welfare development such as improved health care.

Changing educational structures contribute to the total changes seen in LE and may lead to changes in socioeconomic inequalities in LE. In order to assess how educational expansion has impacted the development of LE and the gap in LE between educational groups in the Nordic countries, this study first describes educational differences in LE in old age (65–90 +) and their changes over time (2001–2015), and second examines to what extent the change in national LEs can be attributed to education-specific mortality and to changes in the educational structure. Because of the known sex differences in LE and educational expansion (women started later), all the analyses were stratified by sex.

## Methods

### Materials

This study uses national register data comprising total 65 + populations in four Nordic countries: Denmark, Finland, Norway and Sweden. All the Nordic countries have nationwide registers, which among other things makes it possible to link data on educational attainment with mortality at the individual level (Van Der Wel et al. 2019). The data came from Statistics Denmark (http://www.dst.dk), Statistics Finland (http://www.stat.fi/), Statistics Norway (http://microdata.no) and Statistics Sweden (http://www.scb.se).

Abridged period life tables were calculated based on five-year age groups, 65–69, 70–74, 75–79, 80–84, 85–89 and 90 + , and were closed at age 90. Education was classified into three categories based on the International Standard Classification of Education (ISCED) in each country: 1) low education (ISCED levels 0–2, less than primary, primary or lower secondary education), 2) middle education (ISCED 3–4, upper secondary education), and 3) high education (ISCED 5–8, tertiary education). For Finland, the information on education came from the degree register, which does not distinguish persons with missing information from those with ISCED 0–2. In addition, the educational registers do not cover older populations born before 1922 and 1915 in Denmark and Sweden, respectively. However, this shortcoming has recently been resolved by using a redistribution method for deaths and population exposures with unknown education (Németh et al. 2021). The method is based on a nonparametric approach, assuming that the sum of population-weighted education-specific death rates at each age is equal to the corresponding age-specific death rates for the total population. The re-estimation is performed using extrapolation of education-specific death rate ratios for all age groups after the lowest age at which the share of unknown education in population exceeds 5%. It is assumed that the age- and education-specific death rate ratios in each education group compared to the national death rates fully converge to the total (national) death rate at age 110 (Németh et al. 2021). As the availability of information on the level of education is scarce for the very old before year 2000, the study period started in 2001.

### Analysis

The analysis of changes in LE was performed uniformly across the four countries, based on calculated sex- and education-specific period life tables between 2001 and 2015. The period life tables apply current age-specific mortality rates to a hypothetical cohort and assume that these rates remain constant throughout the life. However, period life tables are influenced by a cohort effect since mortality rates at each age are also a result of the previous mortality in that age cohort (Luy et al. 2020). The period life tables were constructed using mid-year population estimates and the number of deaths for each year under observation. To increase the number of population in the calculations and to avoid annual fluctuation in the estimates, we examined LEs for three five-year time periods: 2001–2005, 2006–2010 and 2011–2015. The final study populations for these three study periods were obtained by summing mid-year population estimates and number of deaths, respectively.

Differences in national LE between 2001–2005 and 2011–2015 were further decomposed into: a) a mortality component (the contributions of mortality changes within each educational group) and b) a compositional component (the contributions of changes in proportions of each educational group) (Andreev et al. 2002; Andreev and Shkolnikov 2012). The estimation of the mortality and compositional contributions was performed in three steps. First, assuming that the national age-specific death rates are equal to a weighted sum of age- and education-specific death rates, LEs at age 65 (LE_65_) for both time points were estimated from a) age- and education-specific death rates and b) age- and education-specific population weights. Second, the decomposition of change in LE_65_ was performed by applying the method of stepwise replacement (Andreev et al. 2002; Andreev and Shkolnikov 2012). This algorithm assumes a stepwise replacement of each cell in the two matrices for the first time period by the corresponding cells from the two matrices in the second time period. The numerical effect of each age-specific replacement (either education-specific death rate or population weight) on the total change in national LE_65_ equals to age-specific contributions of (a) mortality change and (b) compositional change. The (a) mortality component indicates the total change in LE_65_ produced by changes in education-specific death rates under a hypothetical scenario that population composition by education remains fixed at the initial level. The (b) compositional component refers to the additional (to mortality) change in LE_65_ produced by shifts in size of each educational group. The sum of the two components (contributions) is equal to the observed change in national LE_65_.

## Results

### Changes in the size and composition of the study population

**Fig. 1:**
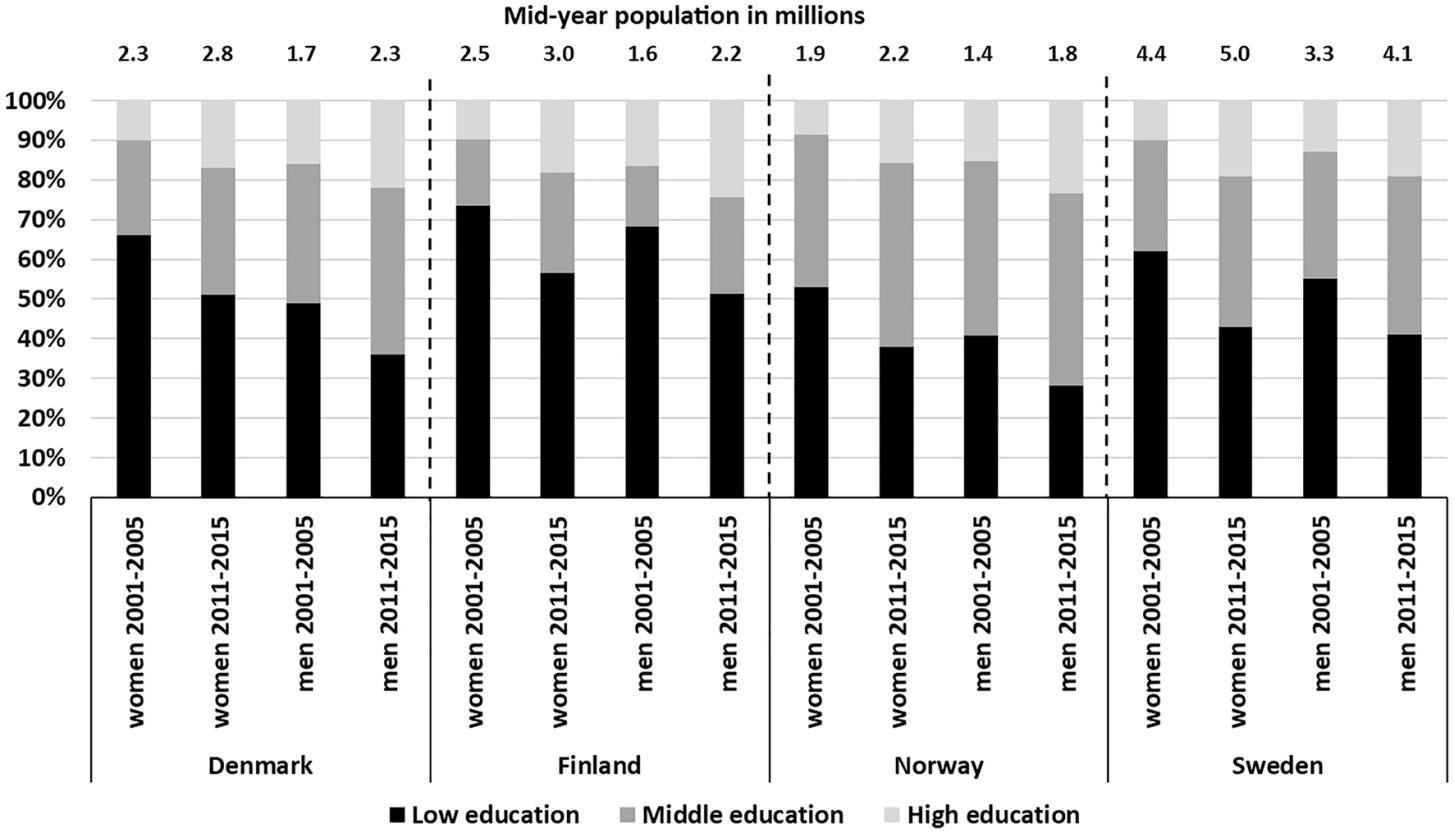
Increase in mid-year population and change in educational structure for 65 + population in Denmark, Finland, Norway and Sweden between 2001–2005 and 2011–2015

The populations aged 65 + increased in all four Nordic countries between 2001 and 2015. The increases were higher in Denmark and Finland than in Norway and Sweden, and higher among men than women. The educational structure also changed during the study period. Norway had the lowest proportion of people with low education and the highest proportion with middle education, whereas Finland had the highest proportion of people with low education and the lowest proportion with middle education both among women and men. Danish men and Swedish women followed Norway having second lowest proportions of people with low education and second highest proportions with middle education. The four countries had different starting points but experienced rather similar proportional changes in the education groups. The proportion of women with low education decreased by 15–19 percentage points (pp) and the proportion of women with high education increased by 7–9 pp over the period. The corresponding figures for men were 13–17 pp decreases in the proportion of those with low education and 6–8 pp increases in the proportion of those with high education (Fig. 1). However, when looking at the relative change in the education groups between 2001 and 2015 (proportion in the education group in 2015/proportion in the education group in 2001), women and men in Norway and Swedish women had the greatest declines in the proportion of people with low education, Finnish women and men had the greatest increases in the middle education group and women in all countries, especially in Sweden, had large increases in the high education group.

### Increases in life expectancy at age 65 (LE_65_) by sex and country

LE_65_ increased in both women and men in all four countries over the study period. Denmark initially had the lowest LE_65_ among women (18.7 years) and men (15.7 years) but recorded the largest increases in LE_65_ (2.0 and 2.1 years, respectively) over time. Sweden had the highest LE_65_ throughout the study period, yet it experienced the smallest increases in LE_65_ (1.0 years for women and 1.6 years for men). Furthermore, women had a higher LE_65_ than men in all countries, but men showed somewhat larger (0.1–0.7 years) increases in LE_65_ during the study period, leading to decreasing gaps in LE_65_ between the sexes (Table 1).

**Table 1:** Increase in life expectancy at age 65 from 2001–2005 to 2011–2015 decomposed into the contributions of changes in mortality and educational structure

	Men		Women	
	Years	%	Years	%
*Denmark*				
Life expectancy 2001–2005	15.7		18.7	
Life expectancy 2011–2015	17.8		20.7	
Life expectancy increase	2.1	100.0	2.0	100.0
Overall mortality effect	1.9	90.5	1.7	86.2
High education	0.3	14.5	0.2	9.6
Middle education	0.7	34.8	0.5	24.0
Low education	0.9	41.2	1.0	52.6
Effect of educational structure	0.2	9.5	0.3	13.8
*Finland*				
Life expectancy 2001–2005	16.2		20.1	
Life expectancy 2011–2015	17.9		21.5	
Life expectancy increase	1.7	100.0	1.4	100.0
Overall mortality effect	1.4	81.9	1.2	83.5
High education	0.2	12.3	0.1	7.8
Middle education	0.2	11.2	0.2	11.7
Low education	1.0	58.5	0.9	63.9
Effect of educational structure	0.3	18.1	0.2	16.5
*Norway*				
Life expectancy 2001–2005	16.6		20.0	
Life expectancy 2011–2015	18.5		21.2	
Life expectancy increase	1.9	100.0	1.2	100.0
Overall mortality effect	1.5	80.9	0.8	70.3
High education	0.2	12.8	0.1	9.9
Middle education	0.7	36.8	0.4	30.2
Low education	0.6	31.3	0.4	30.3
Effect of educational structure	0.4	19.1	0.4	29.7
*Sweden*				
Life expectancy 2001–2005	17.2		20.5	
Life expectancy 2011–2015	18.8		21.5	
Life expectancy increase	1.6	100.0	1.0	100.0
Overall mortality effect	1.4	84.9	0.6	63.2
High education	0.2	12.7	0.1	5.1
Middle education	0.4	27.3	0.2	18.0
Low education	0.7	44.9	0.4	40.0
Effect of educational structure	0.2	15.1	0.4	36.8

### Increases in life expectancy (LE) among women by education, age and country

**Fig. 2:**
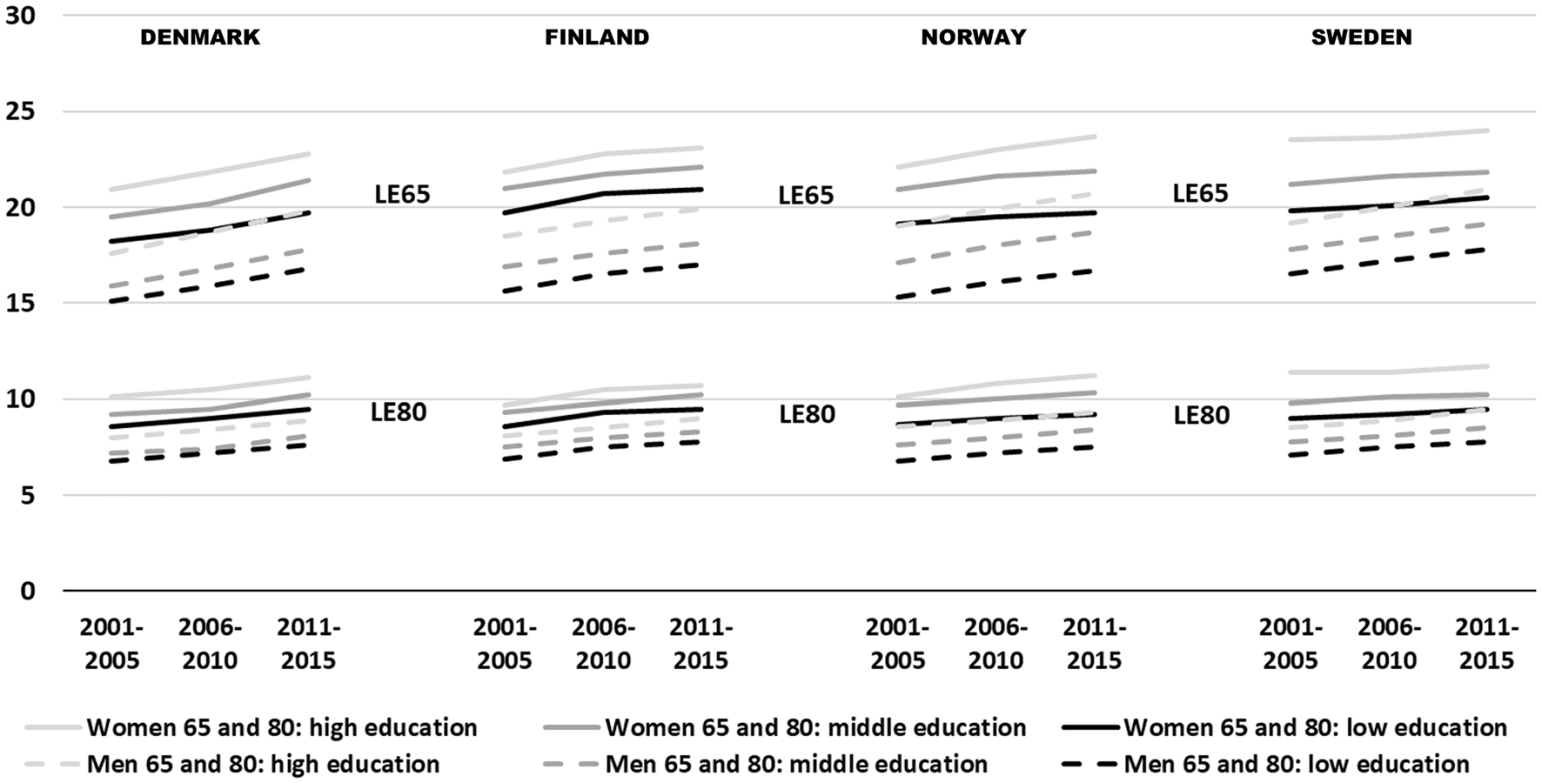
Increase in life expectancy at age 65 and 80 by level of education in Denmark, Finland, Norway and Sweden between 2001–2005 and 2011–2015

In all the three time periods (2001–2005, 2006–2010, and 2011–2015), those with low education had the shortest LE and those with high education the longest LE (Table 2 & Fig. 2). Overall, the educational gaps persisted to the oldest age groups across all countries and showed no signs of stalling with age. In relation to the remaining LE, the gaps tended to increase by age, in all countries except Norway (Supplement Table 1). All educational groups exhibited increases in LE over time, at all ages. LE increase was greater among people with high education in Denmark and Norway but about the same size among low and high education groups in Finland and Sweden. The gap in LE_65_ between those with high and low education was greater in Norway and Sweden compared with Denmark and Finland and increased the most (0.9 years) in Norway over time.

**Table 2:** Life expectancy (LE) in years at age 65, 70, 75, 80, 85 and 90 + for women by level of education in Denmark, Finland, Norway and Sweden in 2001–2005, 2006–2010 and 2011–2015, and over the study period

Period	2001–2005			2006–2010			2011–2015			Change in LE over the period		
Education	low	middle	high	low	middle	high	low	Middle	high	low	middle	high
*Denmark*												
LE_65_	18.2	19.5	20.9	18.8	20.2	21.8	19.7	21.4	22.8	1.5	1.9	1.9
LE_70_	14.6	15.6	16.9	15.2	16.2	17.6	16.0	17.4	18.6	1.4	1.8	1.7
LE_75_	11.5	12.2	13.3	11.8	12.6	13.8	12.5	13.5	14.6	1.0	1.3	1.3
LE_80_	8.6	9.2	10.1	9.0	9.5	10.5	9.5	10.2	11.1	0.9	1.0	1.0
LE_85_	6.3	6.8	7.4	6.6	7.0	7.8	7.1	7.7	8.3	0.8	0.9	0.9
LE_90_	4.7	5.1	5.7	5.0	5.3	6.0	5.4	5.9	6.4	0.7	0.8	0.7
LE_65_ high-low	2.8			3.0			3.1					
*Finland*												
LE_65_	19.7	21.0	21.8	20.7	21.7	22.8	20.9	22.1	23.1	1.2	1.1	1.3
LE_70_	15.7	16.7	17.5	16.6	17.5	18.4	16.9	17.9	18.7	1.2	1.2	1.2
LE_75_	11.9	12.8	13.4	12.7	13.5	14.2	13.0	13.8	14.6	1.1	1.0	1.2
LE_80_	8.6	9.3	9.7	9.3	9.8	10.5	9.5	10.2	10.7	0.9	0.9	1.0
LE_85_	6.0	6.6	6.8	6.4	6.9	7.3	6.6	7.1	7.5	0.6	0.5	0.7
LE_90_	4.1	4.6	4.6	4.4	4.7	5.1	4.4	4.9	5.1	0.3	0.3	0.5
LE_65_ high-low	2.1			2.1			2.2					
*Norway*												
LE_65_	19.1	20.9	22.1	19.5	21.6	23.0	19.7	21.9	23.7	0.6	1.0	1.6
LE_70_	15.3	16.8	17.7	15.7	17.4	18.6	16.0	17.7	19.2	0.7	0.9	1.5
LE_75_	11.7	13.0	13.7	12.1	13.5	14.5	12.4	13.8	15.0	0.7	0.8	1.3
LE_80_	8.7	9.7	10.1	9.0	10.0	10.8	9.2	10.3	11.2	0.5	0.6	1.1
LE_85_	6.3	6.9	7.1	6.5	7.2	7.7	6.6	7.3	8.0	0.3	0.4	0.9
LE_90_	4.6	4.9	4.9	4.7	5.2	5.6	4.7	5.3	5.6	0.1	0.4	0.7
LE_65_ high-low	3.0			3.6			3.9					
*Sweden*												
LE_65_	19.8	21.2	23.5	20.1	21.6	23.6	20.5	21.8	24.0	0.7	0.6	0.5
LE_70_	15.9	17.1	19.2	16.2	17.5	19.3	16.5	17.7	19.6	0.6	0.6	0.4
LE_75_	12.3	13.2	15.2	12.5	13.6	15.2	12.8	13.8	15.5	0.5	0.6	0.3
LE_80_	9.0	9.8	11.4	9.2	10.1	11.4	9.5	10.2	11.7	0.5	0.4	0.3
LE_85_	6.4	7.0	8.3	6.5	7.2	8.3	6.7	7.4	8.5	0.3	0.4	0.2
LE_90_	4.7	5.2	6.3	4.8	5.4	6.3	4.9	5.4	6.5	0.2	0.2	0.2
LE_65_ high-low	3.7			3.5			3.5

### Increases in LE among men by education, age and country

Among men, those with low education had the shortest LE, while those with high education had the longest LE in all three periods (Table 3 and Fig. 2). This applied to all ages in all four countries. Furthermore, all educational groups exhibited increases in LE at all ages over time. LE increase was greater among those with high education than those with low education in all countries except Finland. The gap in LE_65_ between those with high and low education was greatest in Norway and increased by 0.4 years in Denmark, Norway and Sweden over time.

**Table 3:** Life expectancy (LE) in years at age 65, 70, 75, 80, 85 and 90 for men by level of education in Denmark, Finland, Norway and Sweden in 2001–2005, 2006–2010 and 2011–2015, and over the study period

Period	2001–2005			2006–2010			2011–2015			Change in LE over the period		
Education	low	middle	high	low	middle	high	low	middle	high	low	middle	high
*Denmark*												
LE_65_	15.1	15.9	17.6	15.9	16.8	18.7	16.8	17.8	19.8	1.7	1.9	2.2
LE_70_	11.8	12.5	13.9	12.6	13.2	14.8	13.5	14.2	15.8	1.7	1.7	1.9
LE_75_	9.0	9.6	10.6	9.7	10.0	11.3	10.3	10.9	12.1	1.3	1.3	1.5
LE_80_	6.8	7.2	8.0	7.2	7.4	8.4	7.6	8.1	8.9	0.8	0.9	0.9
LE_85_	5.0	5.2	5.8	5.2	5.4	6.1	5.6	5.8	6.4	0.6	0.6	0.6
LE_90_	3.7	3.9	4.4	3.8	4.0	4.5	4.1	4.3	4.9	0.4	0.4	0.5
LE_65_ high-low	2.6			2.8			3.0					
*Finland*												
LE_65_	15.6	16.9	18.5	16.5	17.6	19.3	17.0	18.1	19.9	1.4	1.2	1.4
LE_70_	12.3	13.4	14.6	13.1	14.1	15.4	13.7	14.6	16.0	1.4	1.2	1.4
LE_75_	9.4	10.2	11.1	10.1	10.9	11.7	10.5	11.3	12.2	1.1	1.1	1.1
LE_80_	6.9	7.5	8.1	7.5	8.0	8.5	7.8	8.3	9.0	0.9	0.8	0.9
LE_85_	5.0	5.4	5.7	5.3	5.6	6.0	5.5	5.9	6.3	0.5	0.5	0.6
LE_90_	3.5	3.7	3.9	3.7	3.8	4.3	3.8	4.1	4.4	0.3	0.4	0.5
LE_65_ high-low	2.8			2.8			2.9					
*Norway*												
LE_65_	15.3	17.1	19.0	16.1	18.0	19.9	16.7	18.7	20.7	1.4	1.6	1.7
LE_70_	12.0	13.5	15.1	12.8	14.2	15.8	13.3	14.9	16.6	1.3	1.4	1.5
LE_75_	9.1	10.3	11.6	9.8	10.8	12.0	10.2	11.4	12.7	1.1	1.1	1.1
LE_80_	6.8	7.6	8.6	7.2	8.0	8.9	7.5	8.4	9.3	0.7	0.8	0.7
LE_85_	5.0	5.6	6.3	5.3	5.8	6.4	5.5	6.0	6.7	0.5	0.4	0.4
LE_90_	3.8	4.1	4.5	3.9	4.3	4.6	4.0	4.4	5.0	0.2	0.3	0.5
LE_65_ high-low	3.7			3.8			4.1					
*Sweden*												
LE_65_	16.5	17.8	19.2	17.2	18.5	20.0	17.8	19.1	20.9	1.3	1.3	1.7
LE_70_	12.9	14.0	15.2	13.6	14.6	15.9	14.1	15.3	16.7	1.2	1.3	1.5
LE_75_	9.7	10.7	11.7	10.3	11.1	12.1	10.7	11.7	12.9	1.0	1.0	1.2
LE_80_	7.1	7.8	8.5	7.5	8.1	8.9	7.8	8.5	9.5	0.7	0.7	1.0
LE_85_	4.9	5.4	6.0	5.2	5.7	6.3	5.5	6.0	6.8	0.6	0.6	0.8
LE_90_	3.5	3.9	4.4	3.7	4.1	4.6	3.9	4.4	5.0	0.4	0.5	0.6
LE_65_ high-low	2.7			2.8			3.1					

### Increases in LE_65_ decomposed by the education-specific mortality effect and by educational structure

**Fig. 3:**
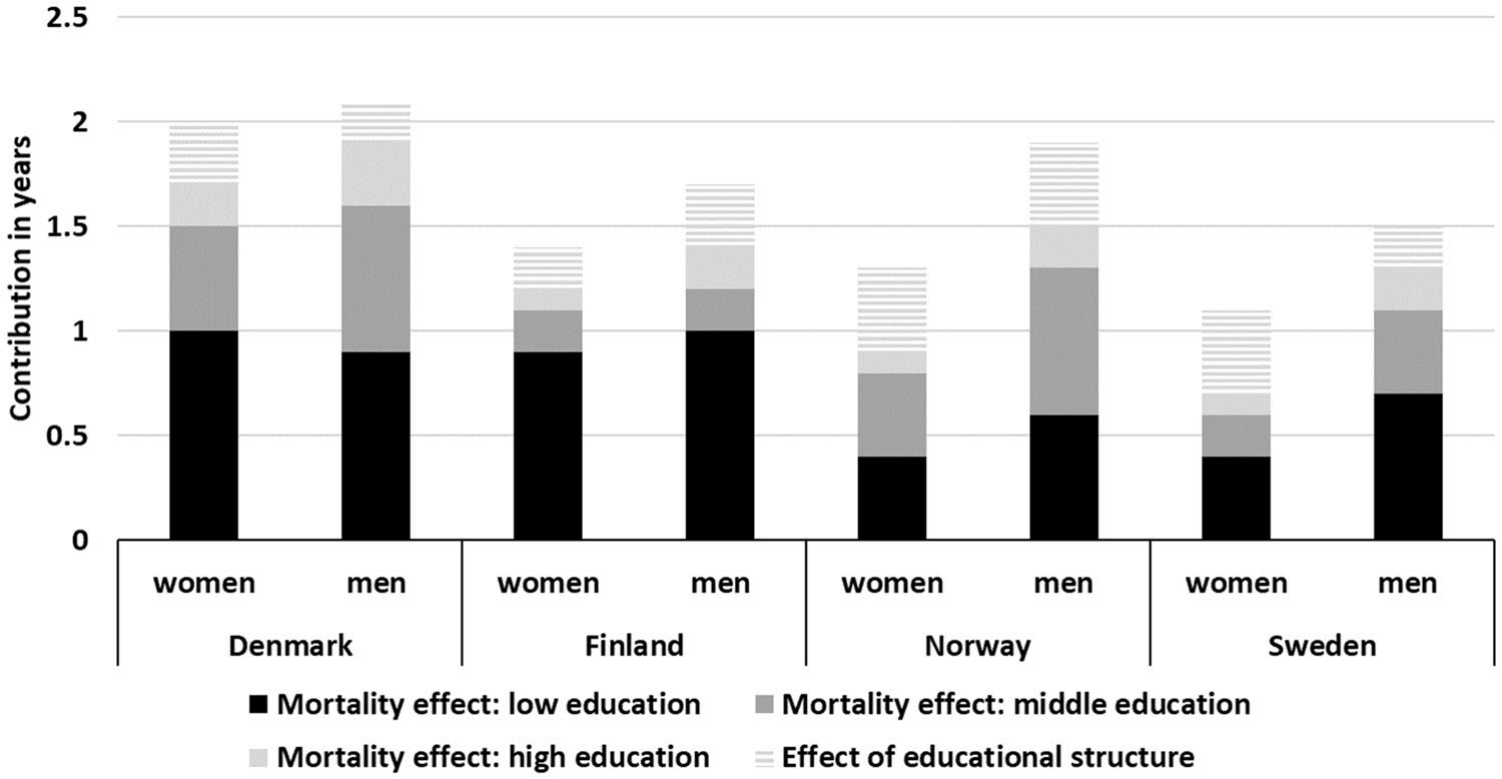
Increase in life expectancy at age 65 between 2001–2005 and 2011–2015 decomposed into the contributions of changes in education-specific mortality and educational structure of the populations in Denmark, Finland, Norway and Sweden

The overall mortality effect contributed more to the increase in LE_65_ than the change in educational structure. Decrease in mortality among those with low education contributed most to the increase in LE_65_, especially in Finland (more than 55%) but also in Denmark and Sweden (more than 40%), which had relatively large groups of people with low education. In Norway, where low and middle education groups were more of a same size, the major contribution to the increase in LE_65_ came from these groups (more than 60%). The proportion of the increase in LE_65_ attributed to the change in the educational structure was 10–20%, but higher in Sweden (30%) and Norway (37%) among women (Table 1 and Fig. 3).

## Discussion

This study examined changes in old age LE by educational attainment in the Nordic countries and decomposed the increases in LE by educational structure and education-specific mortality. Our focus was to study older populations, which increasingly contribute to overall LE. We used national register data comprising total 65 + populations in four Nordic countries, and extended our analyses to more recent years than those covered in earlier studies. Our main findings show that (1) LE increased at all ages and in all educational groups across the four countries and the gap in LE between women and men decreased; (2) inequality in LE between educational groups persisted and increased over time; and (3) most of the gains in LE_65_ could be attributed to the decrease in mortality among those with low education, yet the proportion of these gains explained by educational expansion was as high as 37% among women in Sweden.

Total LE_65_ increased in all four Nordic countries between 2001 and 2015. Danish women and men, starting from initially lower levels, experienced the highest gains in LE, decreasing the differences in LE with the other Nordic countries. Previous studies (Jørgensen et al. 2018) have demonstrated differences in LE between the Nordic countries, and recently more than 60% of the disadvantage among the Danes and Finnish men has been attributed to alcohol- and smoking-related mortality (Östergren et al. 2019).

Women had higher LEs than men, but the pace of the increase was greater among men, resulting in reduced sex differences in LE. Sex differences in LE are typically attributed to combinations of biological factors (e.g. genetic and hormonal), lifestyle and behaviours (e.g. smoking and accidents) and social roles (e.g. occupation and care-seeking) (Oksuzyan et al. 2008). A recent study shows that the major lifestyle-attributable risk factors, smoking, alcohol use and obesity, contributed to the decreasing sex gap in LE as the lifestyle-attributable fraction declined among men but increased among women between 1990 and 2014 (Janssen et al. 2021). Another recent study into causes of death in Sweden showed that the sharper decreases in mortality from ischemic heart diseases and circulatory diseases among men than women accounted for 70% of the reduced sex gap in LE (Sundberg et al. 2018). Hence, it seems that the changes in health behaviours are more favourable among men than women, even though the absolute mortality risks are still higher among men.

Our study showed that in all countries, LE was consistently lowest for those with low education, and there was a pronounced advantage in LE for those with the highest education. Most prior research is based on data limited to working ages or to age groups under 80. In line with earlier studies focusing on older populations (Martelin et al. 1998; Deboosere et al. 2009; Kinge et al. 2015), this study showed persisting and increasing educational gaps in LE also in the oldest age groups. As Rehnberg et al. (2019) show, the divergence or convergence of mortality inequalities vary by age and measure used. In this study, absolute educational inequalities decreased sharply with age but in relation to the remaining LE, inequalities were of the same magnitude or even greater at advanced ages.

The observed changes in LE in the Nordic countries coincided with a substantial compositional change in the educational structures. The decomposition analysis revealed that the change in educational structure contributed to 10–20% of the increase in LE_65,_ but the figure was markedly higher among women in Norway (30%) and Sweden (37%). Overall, the contribution of change in educational structure translates into 0.2–0.4 years increases in LE_65_ between 2001 and 2015. Earlier research has shown rather similar relative contributions of educational expansion to the increase in LE and also a greater advantage among women compared with men, albeit mostly in different countries and at younger ages (Luy et al. 2019).

The four Nordic countries started with different educational distributions, LEs and educational inequalities in LEs, and experienced different developments in them over the study period. Since LE systematically increased in all educational groups across all countries, the change in the educational gap was related to the different pace of gains in LE. For instance, women in Finland and Denmark had large proportions of people with low education who had relatively large increases in LE, which contributed greatly to the total increase in LE. However, women in Norway and Sweden had lower proportion of people with low education who exhibited modest increase in LE, which contributed less to the total increase in LE. As a consequence of decreasing proportion of people with low education, those remaining in the group may have become more negatively selected over time in terms of health and personal characteristics (Mackenbach 2010; Dowd and Hamoudi 2014). In such a case, mortality risk in this group would be increasingly driven by people with health problems and the progress in LE would be harder to achieve. In this study, educational expansion concerned especially the youngest part of the population who have the lowest mortality risk. Thus, the impacts of the greatest reductions in the group with low education are yet to be seen, as the younger people who have experienced educational expansion have not yet reached old age.

Finland was the only country where the educational gap basically remained the same over time. The change in the educational structure was different in Finland, which started with the lowest level of education. Finnish men and women had the greatest increases in the proportion of people with middle education, which in comparison to other education groups, showed the lowest increase in LE. This could imply that the social composition of the middle education group became increasingly heterogeneous i.e. those with less favourable living conditions and thus higher mortality risk had increased access to higher education. At younger ages, the socioeconomic mortality gap has been large in Finland, especially among men (Mäki et al. 2013). However, in this study among populations aged 65 and over, the educational gap in LE among Finnish men was similar with Swedish and Danish men. While the LE in old age does not significantly differ between Finnish men and men in other Nordic countries, it has been shown that the proportion of the birth cohorts reaching old age is lower among men in Finland than in the other Nordic countries (Jørgensen et al. 2018). Hence, mortality selection into old ages might be more pronounced in Finland, and if this effect is stronger among those with low education it might lead to reduced inequalities. Another potential reason for relatively small inequalities in Finland might stem from the fact that it was impossible to separate those with missing information on education from the low education group. If those with missing information had higher than basic education, that might dilute the differences between those with low education and other educational groups.

Denmark and Norway but also Swedish men had great increases in LE among those with high education, which increased the gap between high and low education groups. Education has become a more important source of social division over time, as it has become increasingly associated with career development and assets, which may in turn lead to an accumulation of resources beneficial to health. It is possible that the improved living conditions together with improved access to higher education increased advantage in this educational group. Overall, Norway showed the most pronounced educational gap in LE_65_ among both women (3.0 years in the first and 3.9 years in the last period) and men (3.7 and 4.1 years) and the largest increase in the educational gap in LE_65_ over the study period. Norway is ahead of the other Nordic countries in terms of educational expansion; therefore its Nordic neighbours may well be looking to a future of widening educational inequalities in LE.

A recent stalling of LE progress in the UK has been attributed to cutbacks in funding for health care and social welfare programmes, which has led to an increasing mortality, especially in old age (Hiam et al. 2017). On the other hand, it has been shown that an increase in health care expenditure may contribute to narrowing educational mortality inequalities as it has greater impact on mortality decline among those with low education (Mackenbach et al. 2019). Szebehely and Meagher (2018), and Rostgaard et al. (in this volume) argue that eligibility criteria for access to elder care services such as residential care and home help have tightened in the Nordic countries. Declining coverage of care has in turn disproportionally increased family care among people with fewer resources, while care purchased from the market and paid out-of-pocket by the user has increased among those with more resources. The recent changes in elder care in the Nordic countries and their impact on old age mortality and social inequalities in mortality call for more research.

### Strengths and limitations

The strengths of this study include (1) the use of national register data from four Nordic countries; (2) the use of harmonized life tables and decomposition methods; and (3) the focus on the oldest age groups in analysing inequalities in LE. In addition, educational attainment is an important indicator of socioeconomic conditions since it has far reaching implications for future employment and earnings. Several important limitations of our study should be acknowledged. First, while education is often gained early in life, is stable over time and is likely to precede the onset of diseases and disability for most of the population, it cannot be ruled out that part of the association between education and mortality might be due to health-related selection into low educational attainment. Second, educational attainment is a cohort characteristic, and it should be noted that period life table approach used in this and many other studies provides inferences on mortality of a hypothetical life table cohort reflecting current (period) mortality conditions and does not refer to mortality experience of real cohorts. However, the period mortality rates are affected by the different cohort experiences of the age groups constituting the period life table, including differential access to education. Furthermore, applying cohort life table approach is quite limited for studying the most recent period because such life tables rely on the observed death rates for extinct or at least almost extinct cohorts (Barbieri et al. 2015). Third, it was not possible to completely harmonize the educational classifications across the four countries. For Finland we could not distinguish missing information on education from those with low education, and information on education was missing for older people born before 1922 and 1915 in Denmark and Sweden, respectively. This limitation was partially resolved using a redistribution method for deaths and population exposures with unknown education. The method was validated by sensitivity analyses using Danish register data for recent calendar years and by comparing results from the Németh et al. (2021) study with the results without information on education at advanced ages. Although the adjusted estimates for Denmark and Sweden look plausible, education-specific LE estimates for the most advanced ages beyond 80 rely on extrapolation assumptions and should be treated with caution. More efforts using linkages to other data sources on education such as prior enumeration-based censuses are needed to produce more reliable and statistically robust results.

### Conclusions

The persistent and even growing educational gaps in LE in older ages coincided with increasing LE in all educational groups. Although rising educational levels in the Nordic countries carry potential for further gains in national LE, educational expansion has contributed to uneven gains in LE between education groups. The pronounced educational gaps observed in LE and unequal pace of improvement in older ages in the Nordic countries should be a particular concern because overall LE progress depends on survival and health status at increasingly older ages. The unfavourable trends in LE development, i.e. the large educational mortality gradient and the slower increase in LE compared with the best-performing high-income countries, call for more in-depth research on the potential impact of social policies on maintaining or even increasing health inequalities in welfare states.

## Supplementary Information

Below is the link to the electronic supplementary material.

Supplementary file1 (DOCX 29 KB)

## Data Availability

The data were derived from the Statistics Denmark (http://www.dst.dk), Statistics Finland (http://www.stat.fi/), Statistics Norway (http://microdata.no), and Statistics Sweden (http://www.scb.se).

## Code availability

The datasets generated and analysed during the current study and related codes are available from the corresponding author on reasonable request.
