# Correction to: Changes in socioeconomic differentials in old age life expectancy in four Nordic countries: the impact of educational expansion and education-specific mortality

**DOI:** 10.1007/s10433-022-00711-4

**Published:** 2022-06-28

**Authors:** Linda Enroth, Domantas Jasilionis, Laszlo Németh, Bjørn Heine Strand, Insani Tanjung, Louise Sundberg, Stefan Fors, Marja Jylhä, Henrik Brønnum-Hansen

**Affiliations:** 1 Faculty of Social Sciences (Health Sciences) and Gerontology Research Center, Tampere University, Tampere, Finland; 2 Max Planck Institute for Demographic Research, Rostock, Germany; 3 Department of Physical Health and Ageing, Norwegian Institute of Public Health, Oslo, Norway; 4 The Norwegian National Centre for Ageing and Health, Vestfold Hospital Trust, Tønsberg, Norway; 5 Department of Geriatric Medicine, Oslo University Hospital, Oslo, Norway; 6 Aging Research Center, Karolinska Institutet, Stockholm, Sweden; 7 Stockholm University, Stockholm, Sweden; 8 Department of Public Health, University of Copenhagen, Copenhagen, Denmark

## Correction to: European Journal of Ageing (2022) 19:161–173 10.1007/s10433-022-00698-y

In the original publication of the article, the values in Table 2 were incorrectly placed. The correct Table 2 is given below.

**Table 2:** Life expectancy (LE) in years at age 65, 70, 75, 80, 85 and 90+ for women by level of education in Denmark, Finland, Norway and Sweden in 2001–2005, 2006–2010 and 2011–2015, and over the study period

Denmark	Period	2001–2005			2006–2010			2011–2015			Change in LE over the period		
	Education	Low	Middle	High	Low	Middle	High	Low	Middle	High	Low	Middle	High
	LE_65_	18.2	19.5	20.9	18.8	20.2	21.8	19.7	21.4	22.8	1.5	1.9	1.9
	LE_70_	14.6	15.6	16.9	15.2	16.2	17.6	16.0	17.4	18.6	1.4	1.8	1.7
	LE_75_	11.5	12.2	13.3	11.8	12.6	13.8	12.5	13.5	14.6	1.0	1.3	1.3
	LE_80_	8.6	9.2	10.1	9.0	9.5	10.5	9.5	10.2	11.1	0.9	1.0	1.0
	LE_85_	6.3	6.8	7.4	6.6	7.0	7.8	7.1	7.7	8.3	0.8	0.9	0.9
	LE_90_	4.7	5.1	5.7	5.0	5.3	6.0	5.4	5.9	6.4	0.7	0.8	0.7
	LE_65_ high-low	2.8			3.0			3.1					

Finland	Period	2001–2005			2006–2010			2011–2015			Change in LE over the period		
	Education	Low	Middle	High	Low	Middle	High	Low	Middle	High	Low	Middle	High
	LE_65_	19.7	21.0	21.8	20.7	21.7	22.8	20.9	22.1	23.1	1.2	1.1	1.3
	LE_70_	15.7	16.7	17.5	16.6	17.5	18.4	16.9	17.9	18.7	1.2	1.2	1.2
	LE_75_	11.9	12.8	13.4	12.7	13.5	14.2	13.0	13.8	14.6	1.1	1.0	1.2
	LE_80_	8.6	9.3	9.7	9.3	9.8	10.5	9.5	10.2	10.7	0.9	0.9	1.0
	LE_85_	6.0	6.6	6.8	6.4	6.9	7.3	6.6	7.1	7.5	0.6	0.5	0.7
	LE_90_	4.1	4.6	4.6	4.4	4.7	5.1	4.4	4.9	5.1	0.3	0.3	0.5
	LE_65_ high-low	2.1			2.1			2.2					

Norway	Period	2001–2005			2006–2010			2011–2015			Change in LE over the period		
	Education	Low	Middle	High	Low	Middle	High	Low	Middle	High	Low	Middle	High
	LE_65_	19.1	20.9	22.1	19.5	21.6	23.0	19.7	21.9	23.7	0.6	1.0	1.6
	LE_70_	15.3	16.8	17.7	15.7	17.4	18.6	16.0	17.7	19.2	0.7	0.9	1.5
	LE_75_	11.7	13.0	13.7	12.1	13.5	14.5	12.4	13.8	15.0	0.7	0.8	1.3
	LE_80_	8.7	9.7	10.1	9.0	10.0	10.8	9.2	10.3	11.2	0.5	0.6	1.1
	LE_85_	6.3	6.9	7.1	6.5	7.2	7.7	6.6	7.3	8.0	0.3	0.4	0.9
	LE_90_	4.6	4.9	4.9	4.7	5.2	5.6	4.7	5.3	5.6	0.1	0.4	0.7
	LE_65_ high-low	3.0			3.6			3.9					

Sweden	Period	2001–2005			2006–2010			2011–2015			Change in LE over the period		
	Education	Low	Middle	High	Low	Middle	High	Low	Middle	High	Low	Middle	High
	LE_65_	19.8	21.2	23.5	20.1	21.6	23.6	20.5	21.8	24.0	0.7	0.6	0.5
	LE_70_	15.9	17.1	19.2	16.2	17.5	19.3	16.5	17.7	19.6	0.6	0.6	0.4
	LE_75_	12.3	13.2	15.2	12.5	13.6	15.2	12.8	13.8	15.5	0.5	0.6	0.3
	LE_80_	9.0	9.8	11.4	9.2	10.1	11.4	9.5	10.2	11.7	0.5	0.4	0.3
	LE_85_	6.4	7.0	8.3	6.5	7.2	8.3	6.7	7.4	8.5	0.3	0.4	0.2
	LE_90_	4.7	5.2	6.3	4.8	5.4	6.3	4.9	5.4	6.5	0.2	0.2	0.2
	LE_65_ high-low	3.7			3.5			3.5					

The abstract was incompletely provided on the website. The complete abstract is updated.

The original article has been corrected.

