# Supplementary material for: Changes in socioeconomic differentials in old age life expectancy in four Nordic countries: the impact of educational expansion and education-specific mortality
Source: Eur J Ageing. 2022 Apr 15;19(2):161–73. doi: 10.1007/s10433-022-00698-y (PMC9156635; doi:10.1007/s10433-022-00698-y)
Supplement: Supplementary file 1 — Supplementary file1 (DOCX 29 KB) [file 10433_2022_698_MOESM1_ESM.docx]

Changes in socioeconomic differentials in old age life expectancy in four Nordic countries – The impact of educational expansion and education-specific mortality

European Journal of Ageing

Linda Enroth, Domantas Jasilionis, Laszlo Németh, Bjørn Heine Strand, Insani Tanjung, Louise Sundberg, Stefan Fors, Marja Jylhä, Henrik Brønnum-Hansen

Corresponding author:

Linda Enroth, PhD

Faculty of Social Sciences (Health Sciences) and Gerontology Research Center

Tampere University

Finland

email: linda.enroth@tuni.fi

Supplement table 1. Rate difference (RD) and difference in percentages for life expectancy (LE) between high and low educated at age 65, 70, 75, 80, 85 and 90 among women and men in Denmark, Finland, Norway and Sweden in 2001-2005 and 2011-2015.

|  |  |  | **WOMEN** | | | | **MEN** | | | | |
| --- | --- | --- | --- | --- | --- | --- | --- | --- | --- | --- | --- |
| **Denmark** | Period |  | **2001-2005** | | **2011-2015** | |  | **2001-2005** | | **2011-2015** | |
|  | Education |  | RD | % | RD | % |  | RD | % | RD | % |
|  | LE_65_ |  | 2.7 | 14.8 | 3.1 | 15.7 |  | 2.5 | 16.6 | 3.0 | 17.9 |
|  | LE_70_ |  | 2.3 | 15.8 | 2.6 | 16.3 |  | 2.1 | 17.8 | 2.3 | 17.0 |
|  | LE_75_ |  | 1.8 | 15.7 | 2.1 | 16.8 |  | 1.6 | 17.8 | 1.8 | 17.5 |
|  | LE_80_ |  | 1.5 | 17.4 | 1.6 | 16.8 |  | 1.2 | 17.6 | 1.3 | 17.1 |
|  | LE_85_ |  | 1.1 | 17.5 | 1.2 | 16.9 |  | 0.8 | 16.0 | 0.8 | 14.3 |
|  | LE_90_ |  | 1.0 | 21.3 | 1.0 | 18.5 |  | 0.7 | 18.9 | 0.8 | 19.5 |
| **Finland** | Period |  | **2001-2005** | | **2011-2015** | |  | **2001-2005** | | **2011-2015** | |
|  | Education |  | RD | % | RD | % |  | RD | % | RD | % |
|  | LE_65_ |  | 2.1 | 10.7 | 2.2 | 10.5 |  | 2.9 | 18.6 | 2.9 | 17.1 |
|  | LE_70_ |  | 1.8 | 11.5 | 1.8 | 10.7 |  | 2.3 | 18.7 | 2.3 | 16.8 |
|  | LE_75_ |  | 1.5 | 12.6 | 1.6 | 12.3 |  | 1.7 | 18.1 | 1.7 | 16.2 |
|  | LE_80_ |  | 1.1 | 12.8 | 1.2 | 12.6 |  | 1.2 | 17.4 | 1.2 | 15.4 |
|  | LE_85_ |  | 0.8 | 13.3 | 0.9 | 13.6 |  | 0.7 | 14.0 | 0.8 | 14.5 |
|  | LE_90_ |  | 0.5 | 12.2 | 0.7 | 15.9 |  | 0.4 | 11.4 | 0.6 | 15.8 |
| **Norway** | Period |  | **2001-2005** | | **2011-2015** | |  | **2001-2005** | | **2011-2015** | |
|  | Education |  | RD | % | RD | % |  | RD | % | RD | % |
|  | LE_65_ |  | 3.0 | 15.7 | 4.0 | 20.3 |  | 3.7 | 24.2 | 4.0 | 24.0 |
|  | LE_70_ |  | 2.4 | 15.7 | 3.2 | 20.0 |  | 3.1 | 25.8 | 3.3 | 24.8 |
|  | LE_75_ |  | 2.0 | 17.1 | 2.6 | 21.0 |  | 2.5 | 27.5 | 2.5 | 24.5 |
|  | LE_80_ |  | 1.4 | 16.1 | 2.0 | 21.7 |  | 1.8 | 26.5 | 1.8 | 24.0 |
|  | LE_85_ |  | 0.8 | 12.7 | 1.4 | 21.2 |  | 1.3 | 26.0 | 1.2 | 21.8 |
|  | LE_90_ |  | 0.3 | 6.5 | 0.9 | 19.1 |  | 0.7 | 18.4 | 1.0 | 25.0 |
| **Sweden** | Period |  | **2001-2005** | | **2011-2015** | |  | **2001-2005** | | **2011-2015** | |
|  | Education |  | RD | % | RD | % |  | RD | % | RD | % |
|  | LE_65_ |  | 3.7 | 18.7 | 3.5 | 17.1 |  | 2.7 | 16.4 | 3.1 | 17.4 |
|  | LE_70_ |  | 3.3 | 20.8 | 3.1 | 18.8 |  | 2.3 | 17.8 | 2.6 | 18.4 |
|  | LE_75_ |  | 2.9 | 23.6 | 2.7 | 21.1 |  | 2.0 | 20.6 | 2.2 | 20.6 |
|  | LE_80_ |  | 2.4 | 26.7 | 2.2 | 23.2 |  | 1.4 | 19.7 | 1.7 | 21.8 |
|  | LE_85_ |  | 1.9 | 29.7 | 1.8 | 26.9 |  | 1.1 | 22.4 | 1.3 | 23.6 |
|  | LE_90_ |  | 1.6 | 34.0 | 1.6 | 32.7 |  | 0.9 | 25.7 | 1.1 | 28.2 |
